# Supplementary material for: Visually Driven Neuropil Activity and Information Encoding in Mouse Primary Visual Cortex
Source: Front Neural Circuits. 2017 Jul 21;11:50. doi: 10.3389/fncir.2017.00050 (PMC5519560; doi:10.3389/fncir.2017.00050)
Supplement: Supplementary file 1 [file DataSheet1.docx]

**Supplementary Material**

**
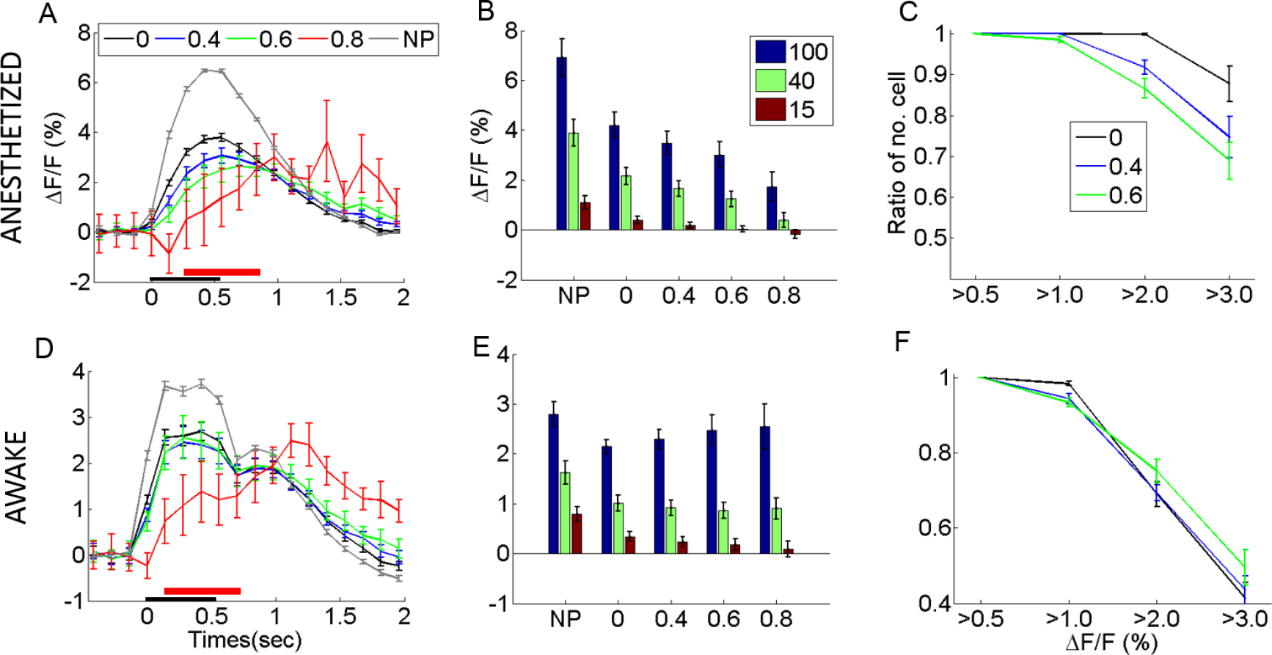
**

**Supplementary Figure 1. Effect of different levels of neuropil contamination correction on visual responses.** Different levels of contamination correction (S) were used to correct the neuropil signal (see Methods), and the corrected ΔF/F response was plotted in the anesthetized (A - C) and awake states (D - F). (**A, D**) A typical example of the time course of the mean response to drifting gratings at 100% contrast, derived from the population of cells in a single field of view (FOV) in the anesthetized state (AN; top) versus the quiet wakefulness state (AW; bottom). The mean time course is corrected by subtracting the mean ΔF/F over 3 frames prior to stimulus onset. Cells shown had a mean response >0.5% to grating stimuli at 100% contrast. Curves with different color reflect different scale corrections (S ranging from 0 to 0.8). ‘NP’ indicates the neuropil signal from annular patches of radii of 7 to 15 µm. Error bars represent the standard error of the mean (S.E.M.). Black and red horizontal bars underlying the time courses correspond to the stimulus-on period and the time period for calculating the mean evoked response of ΔF/F, respectively. Image frame duration was ~130 msec (~7 Hz). The next frame following the onset of the stimulus was taken as t = 0. This means that the first frame at t = 0 sec has a timing jitter of 0-130 msec with respect to the stimulus onset, and thus it is not surprising that sometimes a small evoked response may be seen at t = 0 sec. Note that little change occurs in visually evoked responses for reasonable levels of contamination, i.e. S≤0.6. At higher levels of contamination, however, the cellular response is significantly affected by the correction suggesting that neuropil and cell responses cannot be separated at that stage. (**B, E**) Mean evoked responses to stimuli at 100%, 40%, and 15% contrast, derived from visually responsive cells in 7 AN and 11 AW FOV’s. Cells averaged had ΔF/F responses >0.5% at 100% contrast. ‘NP’ indicates the mean neuropil response. Here, the mean response of ΔF/F is corrected by subtracting the mean ΔF/F of 3 frames (~400 msec) prior to the stimulus onset, and thus slightly negative ΔF/F responses can exist. Note that for contrasts lower than 100% the neuropil contamination correction has a more significant effect. (**C, F**) Number of cells whose mean ΔF/F response to 100% contrast was greater than 0.5, 1.0, 2.0, 3.0% respectively, plotted for S = 0 (black), 0.4 (blue), and 0.6 (green). These numbers are normalized by the total number of cells with a mean response >0.5% under the given correction factor. Note that, in the anesthetized state, the neuropil contamination correction clearly reduces the fraction of cells with high response magnitudes, whereas in the awake state, where responses are overall weaker, this decline does not happen.

**
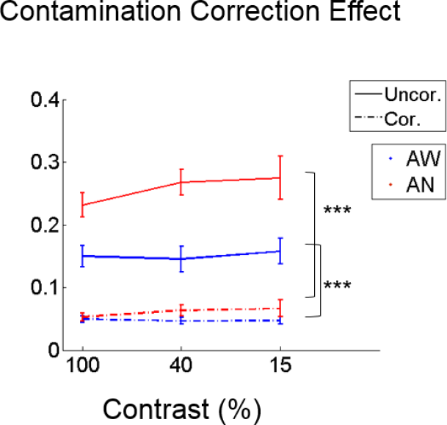
**

**Supplementary Figure 2. Decrease of noise correlations after neuropil contamination correction.** Noise correlations of pairs of cells decrease after correcting for neuropil contamination with S = 0.6. ‘UnC.’ and ‘C.’ represent no correction of neuropil contamination (S = 0) and correction of neuropil contamination with S = 0.6, respectively. *** indicates a significance level of 1e-5.


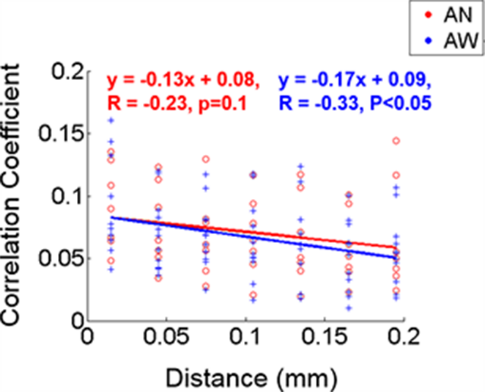


**Supplementary Figure 3. Noise correlation from cellular ΔF/F.** This plot is similar to the one calculated from spike inferred data (see Figure 4A right). This suggests that our analysis on noise correlation is not sensitive to use of a spike-estimation algorithm.


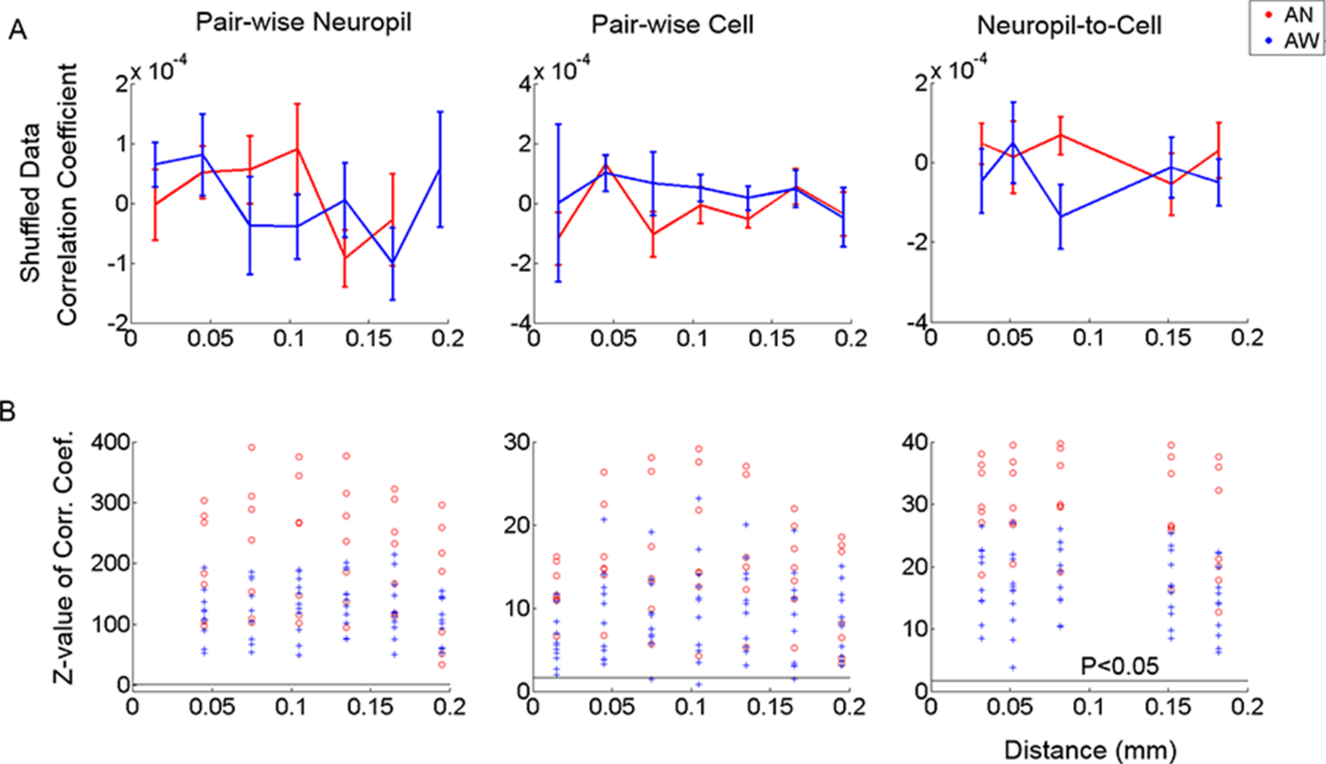


**Supplementary Figure 4**. **Significance of noise correlation.** **(A)** Mean noise correlation from shuffled data across FOVs. After shuffling single-trial responses for each cell within each stimulus condition, noise correlations were calculated. After binning pairs of cells, pairs of neuropil-patches, and pairs of cell and neuropil-patches as a function of distance, noise correlations were averaged within each bin across stimulus conditions and across pairs belonging to each distance bin. This process was performed 1000 times to generate a null distribution at each bin and at each FOV. The plots plotted in (**A**) show the overall mean and SEM of noise correlation across FOVs after averaging noise correlations obtained from the 1000 shuffled datasets. For neuropil-to neuropil, cell-to-cell, and neuropil-to-cell, the shuffled data show very weak correlation (almost 0). **(B)** Z-values of noise correlations from the original data. By using the null distribution generated from the shuffled data, z-values of the original noise correlation coefficients were calculated. The horizontal lines in all three panels reflect a significance level of P<0.05. All data (except for cell-to-cell noise correlations from a single FOV) show statistical significance at levels P<0.05. This suggests that the pair-wise cell and neuropil noise correlation as a function of distance shown in Figure 4 A-B indeed reflect a functional organization of cell and neuropil.


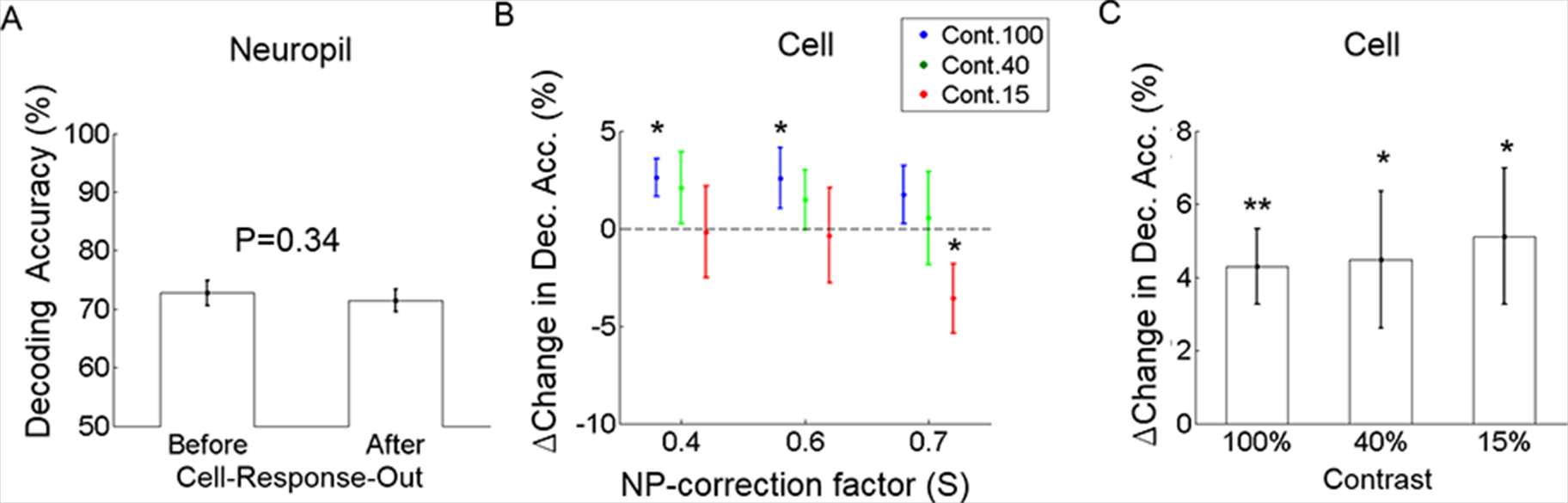


**Supplementary Figure 5.** **Cell versus neuropil-patch decoding performance** **(A)** Decoding accuracy of the neuropil-patch-response vector before and after linear-subtraction of cell responses from adjacent neuropil-patch responses (see Methods for details). The decoding accuracy was averaged across contrasts. Paired t-test was used to report the statistical difference (not significant). The linear subtraction of cell responses from neighboring neuropil-patch responses has no effect on the accuracy of decoding for stimulus direction. This suggests that the neuropil decoding performance did not result from linear contamination of cell responses. (**B**) Decoding Performance as a Function of the Contamination Correction Factor (S). Relative change in the decoding accuracy of the cell population as a function of contamination correction factor (S = 0.4, 0.6, 0.7). The relative change was measured with (decoding accuracy with S – decoding accuracy with S=0)/ (decoding accuracy with S=0). Correcting for neuropil contamination increases decoding accuracy for S=0.4 and 0.6, particularly for high contrasts. At low contrasts (15%) decoding accuracy decreases with S=0.7. * indicates P<0.05 in t-test. Note that the empirically observed correction factors ranged from S =0.4-0.6 in the FOVs imaged. **(C)** Relative change in the decoding performance of *the cell* population after application of the spike estimation algorithm. Specifically, within each FOV, relative decoding accuracy was measured with (‘decoding accuracy using deconvolved spike-responses’ - ‘decoding accuracy using ΔF/F response’) / (‘decoding accuracy using ΔF/F response’ ) only for cell. T-test was used for a statistical test. *, ** refer to P<0.05 and <1e-3. n=18 for all plots.


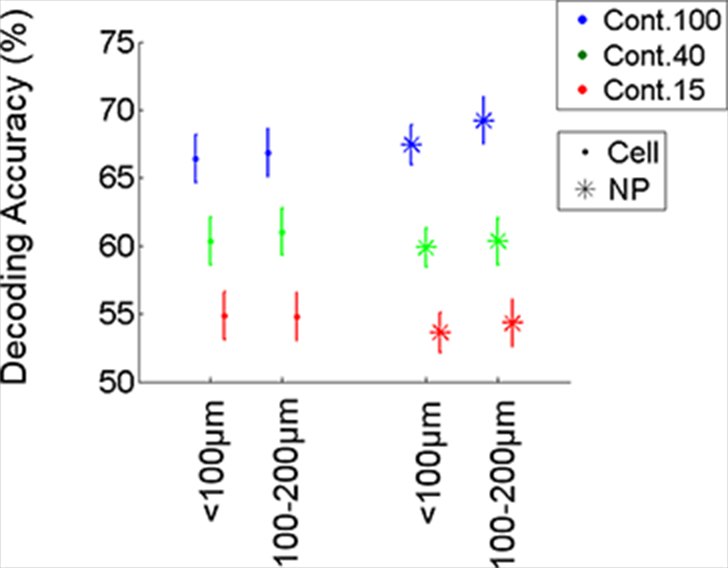


**Supplementary Figure 6. Decoding accuracy as a function of pairwise relative distances.** Decoding accuracy was calculated by randomly selecting groups of 4 cells or neuropil-patches whose pairwise distances were within a given distance range (either <100μm or between 100 to 200 μm),. After averaging the decoding accuracies across groups within each distance bin, the overall mean decoding accuracy was calculated across FOVs. Error bars indicate SEM. There was no difference in decoding accuracy between groups of cells whose relative distances were <100μm, versus groups of cells whose relative distances were greater than 100μm but less than 200μm.
